# Pipersentan: A De Novo Synthetic Endothelin Receptor Antagonist that Inhibits Monocrotaline- and Hypoxia-Induced Pulmonary Hypertension

**DOI:** 10.3389/fphar.2022.920222

**Published:** 2022-06-20

**Authors:** Zeyu Zhang, Chunlei Liu, Yongyi Bai, Xin Li, Xiaojian Gao, Chen Li, Ge Guo, Si Chen, Mingzhuang Sun, Kang Liu, Yang Li, Kunlun He

**Affiliations:** ^1^ Medical Big Data Research Center, Medical Innovation Research Division of Chinese PLA General Hospital, Beijing, China; ^2^ Beijing Key Laboratory of Chronic Heart Failure Precision Medicine, Medical Innovation Research Division of Chinese PLA General Hospital, Beijing, China; ^3^ Department of Cardiology, The Second Medical Center of Chinese PLA General Hospital, Beijing, China; ^4^ Translational Medicine Research Center, Medical Innovation Research Division of Chinese PLA General Hospital, Beijing, China; ^5^ Senior Department of Cardiology, The Sixth Medical Center of Chinese PLA General Hospital, Beijing, China

**Keywords:** endothelin receptor antagonist, Pipersentan, macitentan, pulmonary hypertension, Endothelin (ET-1)

## Abstract

**Background:** Although major advances have been made in the pathogenesis and management of pulmonary arterial hypertension (PAH), the endothelin system is still considered to play a vital role in the pathology of PAH due to its vasoconstrictive action. Endothelin receptor antagonists (ERAs), either as monotherapy or in combination with other drugs, have attracted much attention in the treatment of this lethal disease, and research is continuing.

**Methods:** A novel ERA, pipersentan 5-(1,3-Benzodioxol-5-yl)-6-[2-(5-bromopyrimidin-2-yl)oxyethoxy]-N-(2-methoxyethylsulfamoyl)pyrimidin-4-amine, was recently synthesized and the physicochemical characterizations and the pharmacology both *in vitro* and *in vivo* were studied.

**Results:** This orally administered ERA can both competitively and selectively inhibit the binding of endothelin-1 (ET-1) to its receptors with good physicochemical characteristics. Pipersentan efficaciously antagonized the effects of ET-1 on pulmonary artery smooth muscle cell proliferation, migration and calcium mobilization and effectively improved right ventricular hypertrophy and pulmonary arterial pressure in both monocrotaline- and hypoxia-induced pulmonary hypertension (PH) rat models.

**Conclusions:** This profile identifies pipersentan as a new agent for treating ET-1 system activation-related PH.

## 1 Introduction

Pulmonary arterial hypertension (PAH) is a chronic progressive disease characterized by increased pulmonary artery pressure that severely limits right ventricular function, leading to heart failure and ultimately death. Endothelin-1 (ET-1), a peptide primarily generated by vascular endothelial cells (Barton and Yanagisawa, 2019), plays one of the major roles in the development PAH, that induces the proliferation of vascular smooth muscle cells (SMCs) and thus leads to vasoconstriction. The level of ET-1 is relatively high in patients with PAH and in experimental animal models of pulmonary hypertension (PH) (Belge and Delcroix, 2019; Xiao et al., 2020). Treatment with medications targeting the endothelin pathway has long been one of the most predominant methods in the treatment of PH (Galiè et al., 2015). Bosentan is the first drug in the new class of endothelin receptor antagonists (ERAs) (Williamson et al., 2000).

The functional effects of ET-1 are modulated by two distinct receptors: the ET_A_ and ET_B_ receptors. Under physiological conditions, the ET_A_ receptor is predominantly expressed on SMCs and regulates vasoconstriction, while the ET_B_ receptor is mainly expressed on vascular endothelial cells (ECs) and mediates vasodilation. Ambrisentan has since been discovered as a potent ERA that alleviates PH with higher selectivity toward the ET_A_ receptor rather than the ET_B_ receptor (Galié et al., 2005). However, in pathological conditions such as PH, the ET_B_ receptor is upregulated on SMCs and downregulated on ECs (Iglarz et al., 2015), suggesting that antagonism by dual ERAs may be superior to selective ET_A_ receptor inhibition. The dual ERA macitentan is the most recently approved medicine for PAH and elicits better effects than bosentan through an unclear mechanism (Pulido et al., 2013; Galiè et al., 2019). However, no clinical evidence is available regarding the better efficacy among different types of ERAs (Enevoldsen et al., 2020).

Recommendations for the initial monotherapy were reported in the 2015 ESC/ERS PH guidelines (Galiè et al., 2015). However, if initial monotherapy is chosen, an evidence-based first-line monotherapy cannot be proposed, because head-to-head comparisons between different compounds are not available. Drug selection is affected by a number of factors such as side effects, route of administration, approval status, drug interactions, patient economic status and the preference of the physicians (Galiè et al., 2019). When initial therapy leads to a moderate risk status, combination therapy is recommended. Combinations of sildenafil and macitentan (Pulido et al., 2013), bosentan and riociguat (Ghofrani et al., 2013), and ERAs and selexipag (Sitbon et al., 2015) are the most recommended and have the best supporting evidence. Therefore, ERAs play an essential role in the treatment of PAH either as monotherapies or in combinations with other drugs.

Our goal was to identify a novel ERA with good efficacy and suitability for long-term use. To this end, our discovery process focused on identifying molecules that target both ET_A_ and ET_B_ receptors and with fine physicochemical properties and efficacy. 5-(1,3-Benzodioxol-5-yl)-6-[2-(5-bromopyrimidin-2-yl)oxyethoxy]-N-(2-methoxyethylsulfamoyl)pyrimidin-4-amine, also known as pipersentan, was produced in the tailored screening process and showed a higher affinity toward the ET_A_ receptor rather than the ET_B_ receptor. The pharmacological properties of pipersentan both *in vivo* and *in vitro* was reported in this article with a lower effective dose and a better safety profile than the compared drugs.

## 2 Materials and Methods

### 2.1 Physicochemical Assessment of Pipersentan

*Ionization Constant (pKa):* Both potentiometric titration and ultraviolet spectrophotometry were conducted to determine ionization constant as described previously (Allen et al., 1998).

*Distribution Coefficient (Log D):* The distribution of pipersentan at pH 7.4 between aqueous phosphate buffer and n-octanol was assessed by the flask shaking method as described previously (Iglarz et al., 2008). In brief, pipersentan was dissolved in the organic phase (n-octanol) at a concentration of 0.2%, and then mixed with 67 mM phosphate buffer (pH 7.4) for 15 min by shaking. After separating the two phases via centrifugation, the concentration of pipersentan in each phase was determined *via* high-performance liquid chromatography. D was calculated as the direct quotient of the pipersentan concentrations in the aqueous and organic phases.

### 2.2 Radioligand Binding Assay

Chinese hamster ovary (CHO) cells stably overexpressing the cDNAs for the human ET_A_ and ET_B_ receptors were obtained from Eurofins DiscoverX (Eurofins, United States). Radioligand Binding Assays were conducted by following the kit’s protocols. Membranes were prepared from these cells as described previously (Mihara et al., 1994; Chiou et al., 1997). Briefly, the competition binding assay was conducted in 200 μl of 0.5 mM CaCl_2_, 0.1% bovine serum albumin (BSA) and 50 mM Tris/HCl buffer (pH 7.4) in polypropylene microtiter plates. Membranes containing 0.1 μg ET_A_ or ET_B_ were incubated with increasing concentrations of unlabelled test compounds at 37°C for 2 h with 30 p.m. ^125^I-ET-1 for ET_A_ or at 25°C with 100 p.m. ^125^I-ET-1 for ET_B_, respectively. Minimal and maximal bindings were evaluated in samples with and without 0.1 μM unlabelled ET-1. After incubation, the plates were quantified with a microplate counter (TopCount, Canberra Packard S.A.). The half-maximal inhibitory concentration (IC_50_) value was determined as the concentration required for 50% inhibition of the specific binding of ET-1 by the antagonist.

### 2.3 Calcium Mobilization Assay

Human neuroepithelioma (SK-N-MC) and CHO cells stably overexpressing the cDNAs for the human ET_A_ and ET_B_ receptors were obtained from Eurofins DiscoverX (Eurofins, United States). Human pulmonary artery smooth muscle cells (hPASMCs) were supplied by the American Type Culture Collection (ATCC #PCS-100-023, United States). Calcium mobilization assays was measured by following the kit’s instructions (#90-0091, Eurofins, United States). Cell culture and staining were performed as described previously (Heinroth-Hoffmann et al., 1998; Hayasaki-Kajiwara et al., 1999). Briefly, the cells were incubated with 20 µL Ca NW^PLUS^ Working Reagent at 37°C for 1 h. Then, 10 μl buffer was added, and the mixture was equilibrated at room temperature for 30 min; the cells were analysed with a fluorescent imaging plate reader (FLIPR Tetra, Molecular Devices, United States) with appropriate settings (excitation at 494 nm and emission at 516 nm). In the FLIPR, cells were incubated with the antagonists at increasing concentrations for 2 h and then stimulated by 10 nM ET-1. Calcium mobilization was monitored for 2 min. Percent inhibition was estimated based on the following equation: %Inhibition = 100% x [1-(mean relative fluorescence unit (RFU) of test samples—mean RFU of vehicle controls)/(mean RFU of EC_80_ controls - mean RFU of vehicle controls)]. The IC_50_ value was also calculated.

### 2.4 Binding Assays of cAMP-Related GPCRs

Off-target primary screening of cAMP-related GPCRs was achieved by using a DiscoverX HitHunter cAMP XS + assay (Eurofins, United States) according to the kit’s protocol. Briefly, a standard procedure was used to expand cAMP Hunter cells from freezer stocks. After appropriate time of compound incubation, cAMP XS + ED/CL lysis cocktail (20 µL) was added and incubated for 1 h to generate assay signals. Then, cAMP XS + EA reagent (20 µL) was incubated at room temperature for 3 h, followed by examination with a microplate reader (Envision, PerkinElmer, United States). Percent activity was calculated as follows: %Activity = 100% x (mean relative light unit (RLU) of test samples—mean RLU of vehicle controls)/(mean MAX RLU control ligands—mean RLU of vehicle controls), %Inhibition = 100% x [1-(mean RFU of test samples - mean RFU of vehicle controls)/(mean RFU of EC_80_ controls—mean RFU of vehicle controls)].

### 2.5 Bile Salt Export Pump (BSEP) Inhibition Assay

BSEP inhibition assay was performed with BSEP-Hi5-VT (Solvo Biotechnology, United States) by following the manufacturer’s protocol. Briefly, compounds with decreasing concentrations were placed into microtiter plates. After incubation with certain buffers and filtration, taurocholic acid was detected by LC-MS/MS, and the IC_50_ value was calculated.

### 2.6 Cell Proliferation and Cytotoxicity Analysis

Cell proliferation and death levels were determined using the Cell Proliferation and Cytotoxicity Assay Kit (#CA1210, Solarbio, China) and Cytotoxicity Lactate Dehydrogenase Detection Kit (#BC0685, Solarbio, China), respectively, according to the kit’s protocols. Briefly, hPASMCs (5,000 cells for each well) were grown in 96-well plates for 6 h, starved for 18 h with cell culture medium containing 0.5% fetal bovine serum (FBS, #10100147, Gibco, United States), treated with dimethyl sulfoxide (DMSO), pipersentan (0.1-100 μM), macitentan (0.1-100 μM), staurosporine (0.2 μM, #HY-15141, MedChemExpress, United States), or Triton (0.1%), and cultured with or without ET-1 (1 μM) for 24 h. Adherent cells were used to detect DNA synthesis, and the supernatants were collected for analysis of the enzymatic activity of lactate dehydrogenase (LDH). An automatic microplate reader (Varioskan LUX, Thermo Scientific, United States) was used to read the 96-well plates.

### 2.7 Migration Assay

Transwell chambers (8 μm pore, Corning, United States) were used for migration assays. hPASMCs (50,000 cells for each well) were seeded into the upper chambers with cell culture medium containing 0.5% FBS. The lower chambers were placed in 24‐well plates with cell culture medium containing 10% FBS with or without ET-1 (1 μM), and added with DMSO, pipersentan (0.1–10 μM) or macitentan (10 μM). After incubation for 24 h, the cells on the lower side of the membrane were fixed with 4% paraformaldehyde and stained with crystal violet staining solution (#G1063, Solarbio, China). Images were captured by an inverted fluorescence microscope (Nikon). The cells were then resuspended in DMSO, and the absorbance was measured using 590 nm as a reference wavelength.

### 2.8 *In Vivo* Pharmacology of Animals

SD rats (males, 220–250 g) were supplied by the Experimental Animal Center of the Chinese PLA General Hospital (Beijing, China). The rats were maintained at 22–25°C under a 12:12-h light/dark cycle and were given free access to water and food. The bedding was changed twice a week. All experimental procedures were approved by the Animal Ethics Committee of the Chinese PLA General Hospital.

### 2.9 Effects on Endothelin-1 Plasma Concentrations

Sublingual blood samples for ET-1 plasma measurements were collected under 2.5% isoflurane anesthesia from male SD rats before administration and 0.5 and 1 h after administration of pipersentan at increasing concentrations (1, 3, 10 mg/kg) or vehicle administration by oral gavage (*n* = 6 for each group). The ET-1 concentration was measured by using an Endothelin-1 Quantikine ELISA Kit (#DET100, R&D Systems, United States) according to the manufacturer’s instructions.

### 2.10 Monocrotaline (MCT)-Induced Pulmonary Hypertension and Treatment

The rats were randomly divided into the following six groups (*n* = 12 for control group and *n* = 20 for MCT groups): 1) a control group treated with vehicle, 2) an MCT-induced PH group treated with vehicle, 3) an MCT-induced PH group treated with macitentan (30 mg/kg), 4) an MCT-induced PH group treated with 5 mg/kg pipersentan, 5) an MCT-induced PH group treated with 15 mg/kg pipersentan, and 6) an MCT-induced PH group treated with 30 mg/kg pipersentan. The animals were subcutaneously injected with saline or 60 mg/kg MCT (#C2401, Sigma-Aldrich), and treatments by daily oral gavage started on day 14 and were continued during the subsequent 2 weeks.

### 2.11 Hypoxia-Induced Pulmonary Hypertension and Treatment

The rats were randomly assigned to the following six groups (*n* = 12 for each group): 1) a control group treated with vehicle, 2) a hypoxia-induced PH group treated with vehicle, 3) a hypoxia-induced PH group treated with macitentan (30 mg/kg), 4) a hypoxia-induced PH group treated with 5 mg/kg pipersentan, 5) a hypoxia-induced PH group treated with 15 mg/kg pipersentan, and 6) a hypoxia-induced PH group treated with 30 mg/kg pipersentan. The animals were placed in a hypobaric hypoxia chamber (with a barometric pressure of approximately 380 mmHg) or in normal air (with a barometric pressure of approximately 760 mmHg) for 28 days. Treatments by daily oral gavage started on day 14 and were continued during the subsequent 2 weeks.

### 2.12 Ultrasonic Cardiogram, Hemodynamics, and Right Ventricular Hypertrophy Index (RVHI)

Rats were anesthetized with isoflurane gas, and transthoracic ultrasonic cardiogram was conducted using a Vevo 2100 system (VisualSonics, Canada) coupled to a 30-MHz transducer (Dromparis et al., 2013; Weiss et al., 2019). Briefly, rats were positioned in the supine position on a heating platform with electrodes taped to all legs for heart rate monitoring after anesthesia. To reduce ultrasound attenuation, the chest of each rat was shaved and treated with a chemical hair remover. After spreading the prewarmed ultrasound gel over the chest wall, and the tricuspid annular plane systolic excursion (TAPSE) and right ventricular wall thickness (RVWT) were determined.

Vascular pressures were assessed invasively with Millar catheters as previously described (Shatat et al., 2014; Ogoshi et al., 2018). Briefly, rats were anesthetized with 30 mg/kg pentobarbital sodium (3%) via intraperitoneal injection under sterile conditions. Each animal was given breathing assistance with a rodent ventilator (Kent Scientific, United States) after tracheotomy. A Millar SPR 838 pressure-volume catheter (ADInstruments, United States) was inserted into the right ventricle (RV) through a parasternal incision, and then advanced into the pulmonary artery. MPVS Ultra system equipped with a PowerLab data acquisition system (ADInstruments, United States) was used for pressure measurements. The right ventricular systolic pressure (RVSP) and mean pulmonary artery pressure (mPAP) was then estimated.

After hemodynamic measurements were performed, and the hearts were harvested. After washing twice with ice-cold saline, the weights of RV and left ventricle (LV) with septum (LV + S) were measured, and the RV/(LV + S) mass ratio was calculated.

### 2.13 Histopathological Analysis

The medial wall thickness (MWT) was determined by histopathological analysis as previously described (Sutendra et al., 2010; Dromparis et al., 2013). The upper left lungs were fixed by inflation with 10% formalin, embedded in paraffin, and sectioned for histology. Then, the lung tissue sections (5 μm) were incubated with 5% BSA for 30 min at room temperature after antigen retrieval and hematoxylin-eosin (HE) staining was performed to determine the morphological effects. Images were acquired using Leica Application Suite software and analyzed with Image-Pro Plus 6.0 software. In each rat, 20–30 vessels with diameters of 50–100 μm were identified and measured at their two ends to determine the shortest external diameter (2 × wall thickness/external diameter); the average thickness was considered the MWT.

### 2.14 Compounds

ET-1 and ^125^I-ET-1 were obtained from Eurofins DiscoverX (Eurofins, United States). Macitentan and pipersentan were synthesized by WuXi AppTec (WuXi AppTec, China).

### 2.15 Statistics

Statistical analysis was performed with GraphPad Prism eight software, and all values are expressed as mean ± standard deviation unless otherwise stated. The homogeneity of variance and normality were tested before performing parametric tests. The mean values between groups were compared with unpaired Student’s t-tests or ANOVA with Tukey’s post-hoc tests, whenever appropriate. Level of statistical significance was set as *p* < 0.05.

## 3 Results

### 3.1 Chemical Synthesis of Pipersentan

**FIGURE 1:**
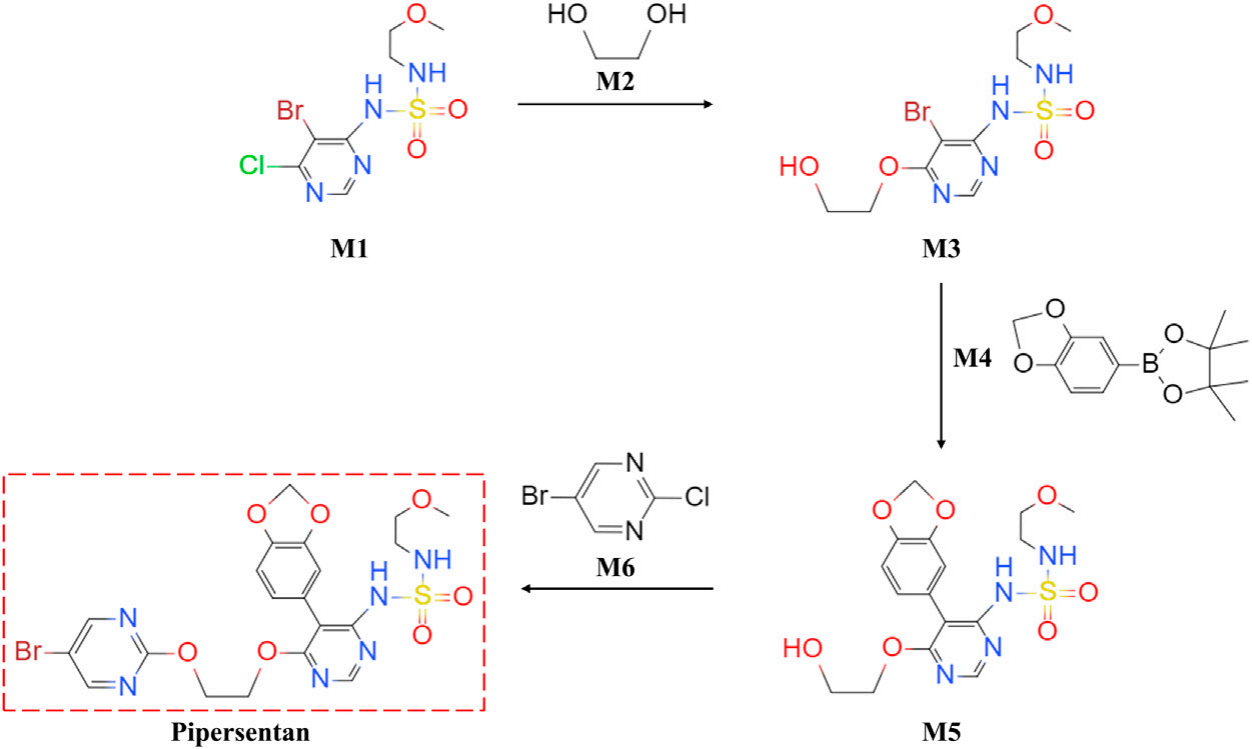
Chemical Synthesis of Pipersentan. Pipersentan was synthesized in three steps. First, the commercially available 1-(5-bromo-6-chloropyrimidin-4-yl)-N-(2-methoxyethyl)azanesulfonamide (**M1**) underwent nucleophilic substitution with ethylene glycol (**M2**) to give the intermediate 1-{5-bromo-6-[(2-hydroxyethyl)oxy]pyrimidin-4-yl}-N-(2-methoxyethyl)azanesulfonamide (**M3**). Then, the intermediate 1-[5-(benzo[d][1,3]dioxol-5-yl)-6-[(2-hydroxyethyl)oxy]pyrimidin-4-yl]-N-(2-methoxyethyl)azanesulfonamide (**M5**) was obtained by the Suzuki coupling reaction from **M3** and 2-(benzo[d][1,3]dioxol-5-yl)-4,4,5,5-tetramethyl-1,3,2-dioxaborolane (**M4**). Finally, **pipersentan** was obtained through the nucleophilic substitution reaction from **M5** and 5-bromo-2-chloropyrimidine (**M6**).

Pipersentan was synthesized in three steps. First, the commercially available 1-(5-bromo-6-chloropyrimidin-4-yl)-N-(2-methoxyethyl)azanesulfonamide (M1) underwent nucleophilic substitution with ethylene glycol (M2) to give the intermediate 1-{5-bromo-6-[(2-hydroxyethyl)oxy]pyrimidin-4-yl}-N-(2-methoxyethyl)azanesulfonamide (M3). Then, the intermediate 1-[5-(benzo[d][1,3]dioxol-5-yl)-6-[(2-hydroxyethyl)oxy]pyrimidin-4-yl]-N-(2-methoxyethyl)azanesulfonamide (M5) was obtained by the Suzuki coupling reaction from M3 and 2-(benzo[d][1,3]dioxol-5-yl)-4,4,5,5-tetramethyl-1,3,2-dioxaborolane (M4). Finally, pipersentan was obtained through the nucleophilic substitution reaction from M5 and 5-bromo-2-chloropyrimidine (M6). (Figure 1). For details, please see Supplementary Figure S1.

### 3.2 Physicochemical Parameters of Pipersentan

Pipersentan exhibited a pKa value of 6.46 and an affinity for the lipophilic phases, as indicated by the distribution of 10 to 1 (Table 1).

**TABLE 1:** Physicochemical parameters of pipersentan.

	Distribution Coefficient D (n-Octanol/Aqueous Buffer)	Log D	pKa
***Pipersentan***	10:1	1	6.46

### 3.3 *In Vitro* Receptor Selectivity and Functional Inhibitory Potency

The affinity of pipersentan and macitentan for ET receptors was evaluated in the microsomal membranes of CHO cells stably overexpressing human ET_A_ or ET_B_ receptors. Pipersentan suppressed the binding of ^125^I-ET-1 toward recombinant ET_A_ receptor with average IC_50_ values of 4.44 nM, which was similar to that of macitentan (4.72 nM), a dual ERA. The suppression activity of pipersentan against the ET_B_ receptor was a little lower, with an IC_50_ of 4.53 μM, than that of macitentan (2.44 μM). In a functional *in vitro* assay, pipersentan completely inhibited the high intracellular calcium induced by ET-1 in recombinant CHO and SK-N-MC cells. The antagonistic activity of pipersentan against the ET_A_ receptor was very high, with an average IC_50_ of 1.6 nM, which was similar to that of macitentan (0.52 nM). The antagonistic activity of pipersentan against the ET_B_ receptor was much lower, with an IC_50_ of 32.0 μM, than that of macitentan (3.40 μM). These results indicated that pipersentan was a dual ERA that had higher selectivity toward the ET_A_ receptor rather than the ET_B_ receptor (Table 2).

**TABLE 2:** *In Vitro* Receptor Selectivity and Functional Inhibitory Potency (*n* = 3).

	Target	Radioligand Binding assay Calcium Mobilization assay	
	(cDNA)	(IC_50_)	(IC_50_)
***Macitentan***	*ET*_*A*_	4.72 ± 0.89 nM	0.52 ± 0.11 nM
	*ET*_*B*_	2.44 ± 0.77 μM	3.40 ± 1.85 μM
***Pipersentan***	*ET*_*A*_	4.44 ± 0.98 nM	1.60 ± 1.02 nM
	*ET*_*B*_	4.53 ± 0.58 μM^a^	32.0 ± 11.0 μM^a^

a *p* < 0.05 vs. Macitentan, unpaired Student’s t-tests.

To evaluate its selectivity, pipersentan (10 μM) was tested in a panel of 24 GPCR cAMP modulation assays. The antagonistic effect of pipersentan on EDNRA (ET_A_) at 10 μM showed a 99.1% response rate. The agonistic response rate of cannabinoid receptor 1 (CNR1) was 72.6%, and the other response rates were less than 70% (Supplementary Table S1).

The inhibitory effect of pipersentan on BSEP (IC_50_ = 43.8 μM) was significantly milder than that of macitentan (IC_50_ = 0.47 μM), suggesting that the inhibitory effect of pipersentan on bile salt transport was smaller than that of macitentan (Supplementary Figure S2).

### 3.4 Effects of Pipersentan on Human Pulmonary Artery Smooth Muscle Cells Proliferation, Migration and Calcium Mobilization

**FIGURE 2:**
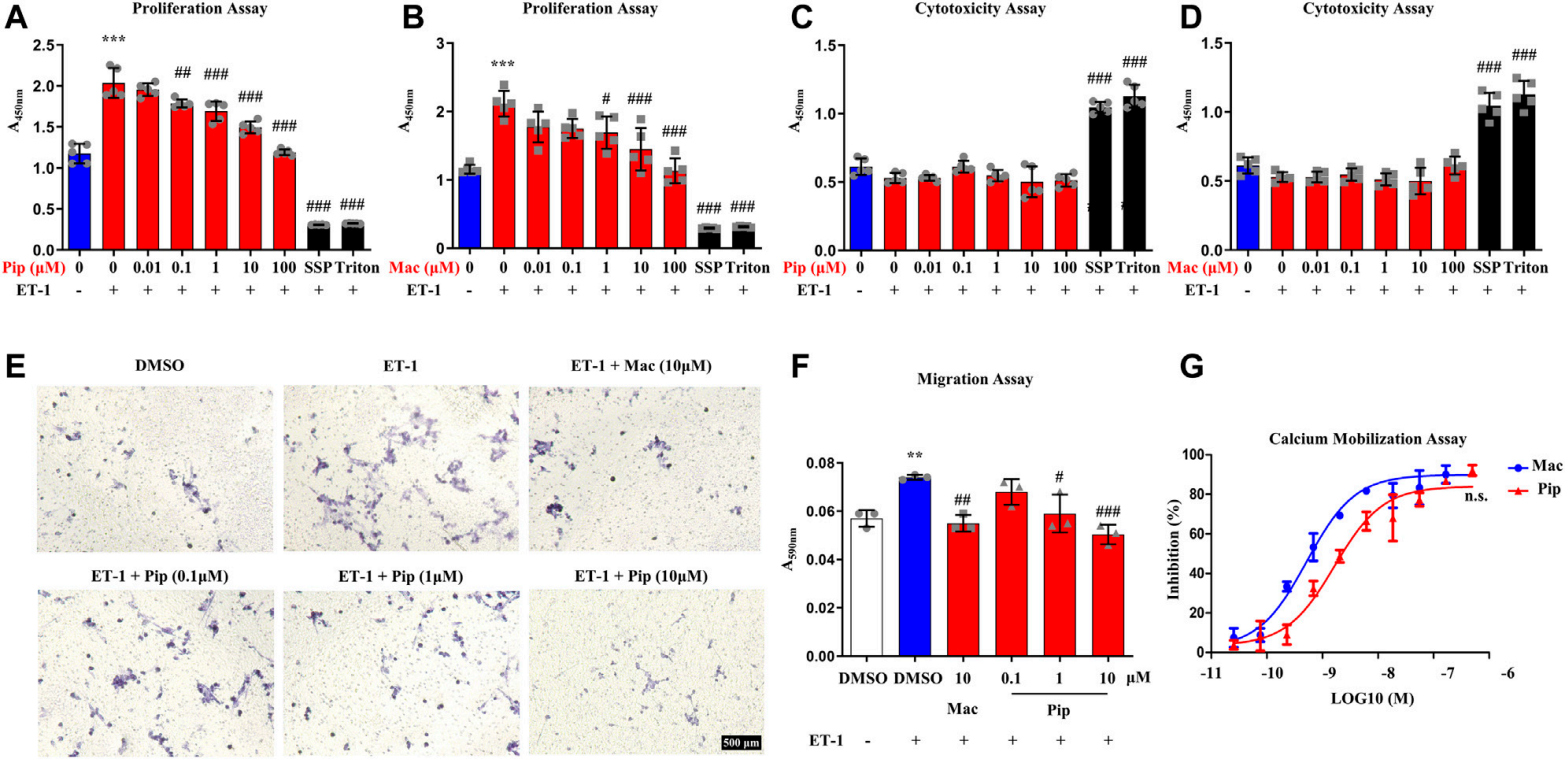
Effects of Pipersentan on hPASMC Proliferation, Migration and Calcium Mobilization **(A–D)** hPASMCs were treated with DMSO, Pipersentan (0.01–100 μM), Macitentan (0.01–100 μM), SSP (0.2 μM) or Triton and exposed to DMSO or ET-1 (1 μM) for 24 h. **(A,B)** Proliferation was determined by detecting DNA synthesis in adherent cells (*n* = 5). **(C,D)** Cytotoxicity was determined by detecting LDH released into the cell culture medium (*n* = 5) **(E,F)** hPASMCs were treated with DMSO, Macitentan (10 μM) or Pipersentan (0.1–10 μM) and exposed to DMSO or ET-1 (1 μM) for 24 h. **(E)** Representative images of migrated cells (scale bars, 500 μm, Original magnification: 200). **(F)** Quantification of the crystal violet levels (*n* = 3) **(G)** The antagonistic activity against hPASMCs was determined by detecting the intracellular calcium mobilization (*n* = 3). The IC_50_ of Macitentan and Pipersentan was 0.51 ± 0.56 nM and 1.66 ± 0.51 nM. The data are presented as the mean ± standard deviation, ^***^ *p* < 0.001 vs. ET-1 (-); ^###^ *p* < 0.001, ^##^ *p* < 0.01, ^#^ *p* < 0.05 vs. ET-1 (+), n. s., not significant, ANOVA with Tukey’s post hoc in **(A–F)**; n. s., not significant, unpaired Student’s t-tests in **(G)**; SSP, staurosporine; Pip, Pipersentan; Mac, Macitentan; ET-1, endothelin-1; LDH, lactate dehydrogenase; DMSO, dimethyl sulfoxide.

Previous studies have shown that abnormal proliferation and migration of PASMCs are related to vascular remodelling in PH (He et al., 2018). hPASMCs were treated with pipersentan at a series of concentrations and exposed to ET-1 for 24 h, and macitentan was used as positive control. The proliferation of hPASMCs decreased with pipersentan treatment in a concentration-dependent manner, but no marked differences in cytotoxicity were found (Figures 2A–D). To explore the effects of pipersentan on hPASMC migration, transwell migration assays were conducted. Compared with DMSO, cell migrations treated with pipersentan and macitentan showd no significant differences without ET-1 exposure (Supplementary Figure S3). Cell migration was significantly increased after ET-1 stimulation for 24 h, whereas pipersentan attenuated the increases in migration with a concentration-dependent effect (Figures 2E,F). There was no significant difference between the pipersentan and macitentan goups at 10 μM. The antagonistic activity of pipersentan against hPASMCs was also determined by detecting intracellular calcium mobilization. Pipersentan inhibited the calcium flux induced by ET-1 with an average IC_50_ value of 1.66 nM, which was similar to that of macitentan (0.51 nM) (Figure 2G). These results revealed that pipersentan could efficaciously antagonize the effects of ET-1 on hPASMC proliferation, migration and calcium mobilization.

### 3.5 Effects of Pipersentan on Endothelin-1 Plasma Concentrations

The endothelin receptor antagonism of pipersentan was tested in rats by measuring plasma ET-1 concentrations. Compared with the vehicle, pipersentan at the dose of 10 mg/kg significantly increased the level of ET-1 at 0.5 and 1 h after administration, and the effects presented a dose-dependent trend, confirming the blockade of endothelin receptors (Supplementary Figure S4).

### 3.6 Pipersentan Improved Cardiopulmonary Function and Remodeling in Monocrotaline-Induced Pulmonary Hypertension Rats

**FIGURE 3:**
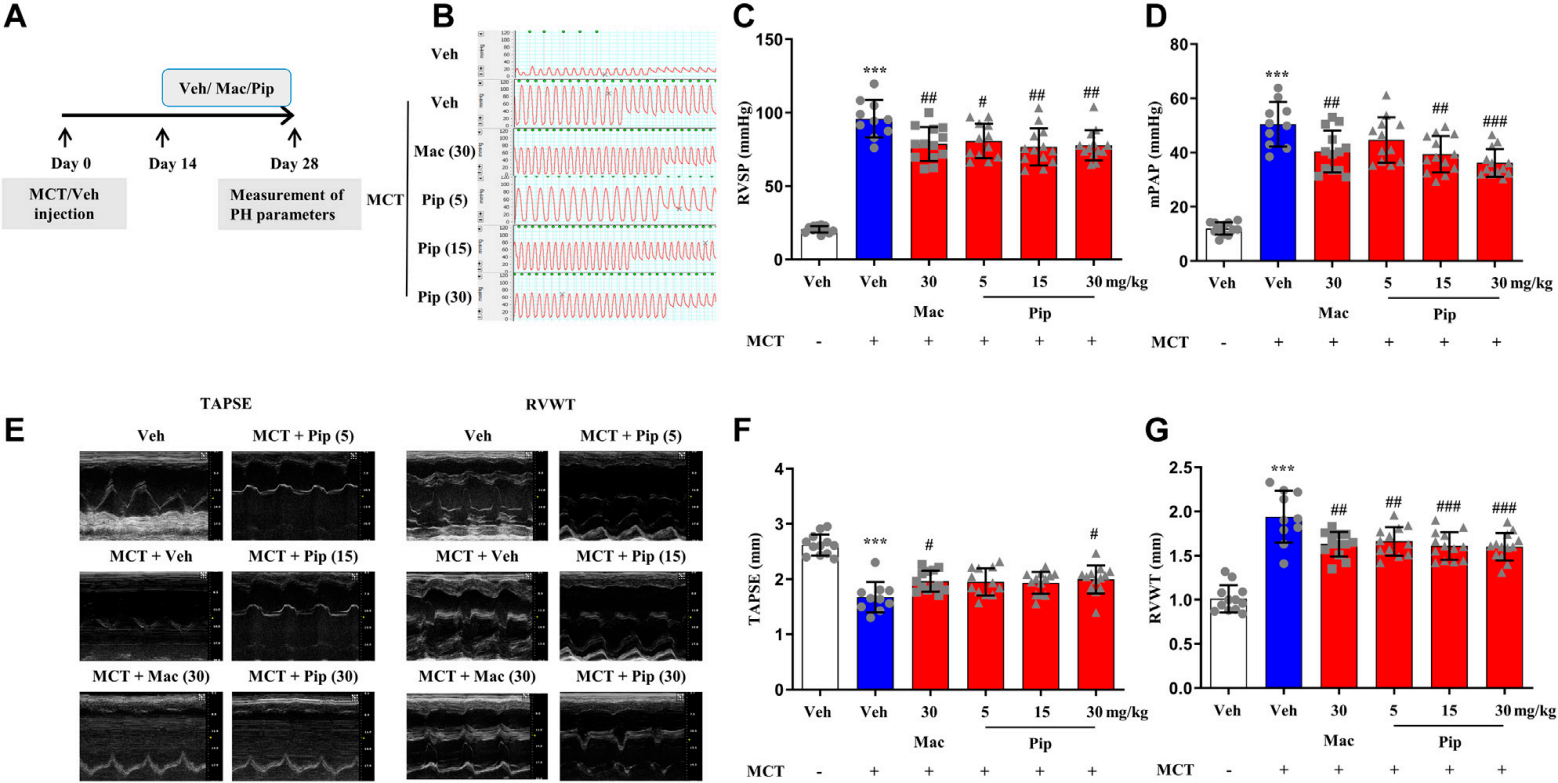
Pipersentan Improved Cardiopulmonary Function in Rats with MCT-induced PH (n = 12, 10, 13, 12, 13 and 13 for each group) **(A)** Diagrammatic sketch of the animal protocol. Rats were subcutaneously injected with MCT at a dose of 60 mg/kg or with vehicle. Treatments by daily oral gavage started on day 14 and were continued for 2 weeks. Measurement of PH parameters was performed on day 28 **(B–D)** Hemodynamic measurements. **(B)** Representative images. **(C)** RVSP. **(D)** mPAP **(E–G)** UCG analyses. **(E)** Representative images. **(F)** TAPSE. **(G)** RVWT. The data are presented as the mean ± standard deviation, ^***^ *p* < 0.001 vs. veh, ^###^ *p* < 0.001 vs. MCT + veh, ^##^ *p* < 0.01 vs. MCT + veh, ^#^ *p* < 0.05 vs. MCT + veh, ANOVA with Tukey’s post hoc test. Veh, vehicle; Mac, macitentan; Pip, Pipersentan; RVSP, right ventricular systolic pressure; mPAP, mean pulmonary artery pressure; UCG, ultrasonic cardiogram; TAPSE, tricuspid annular plane systolic excursion; RVWT, right ventricular wall thickness.

**FIGURE 4:**
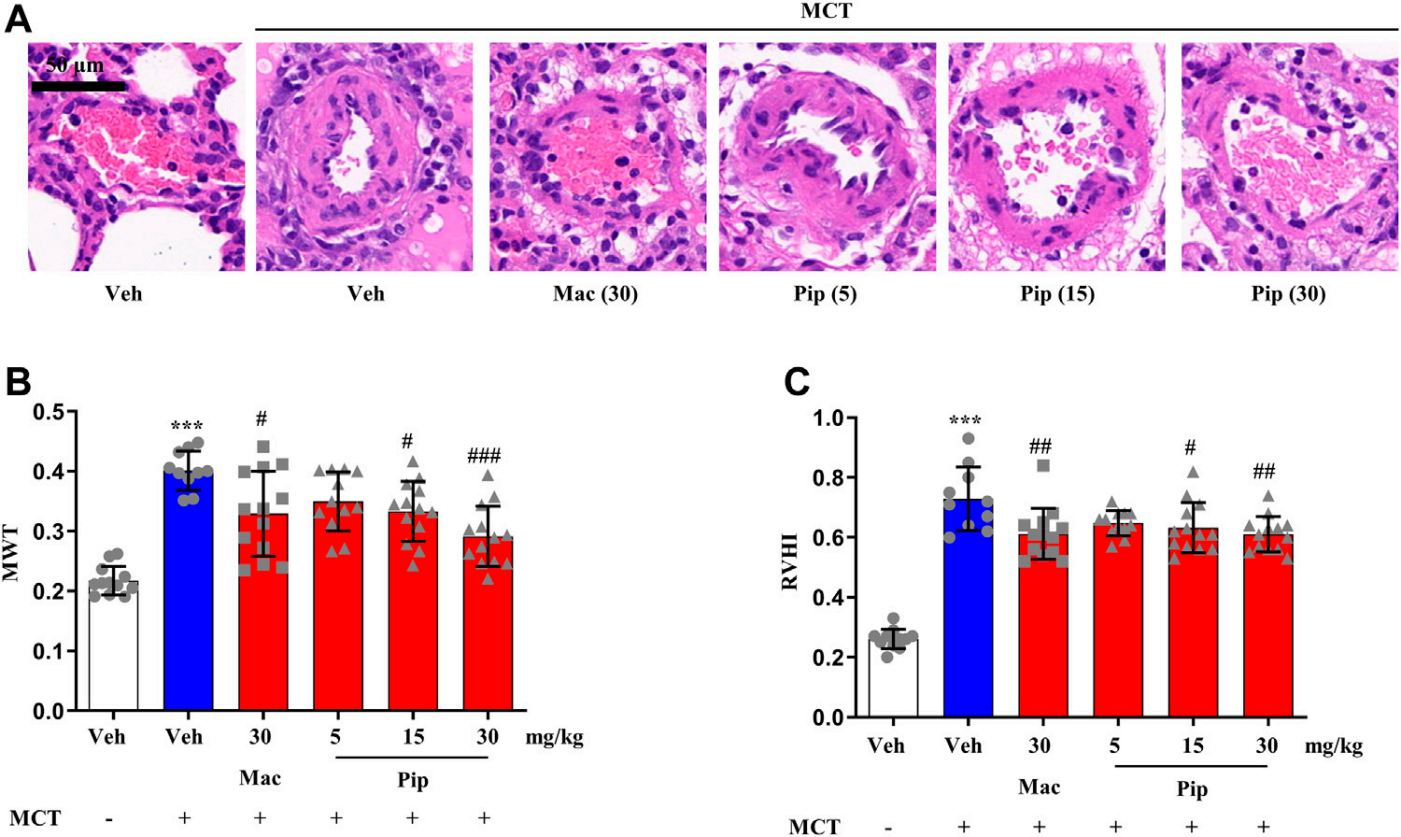
Pipersentan Improved Cardiopulmonary Remodeling in Rats with MCT-induced PH (*n* = 12, 10, 13, 12, 13 and 13 for each group) **(A,B)** Histopathological analysis. **(A)** Representative images of pulmonary arterioles (scale bars, 50 μm, Original magnification: 200). **(B)** MWT **(C)** RVHI. The data are presented as the mean ± standard deviation, ^***^ *p* < 0.001 vs. veh, ^###^ *p* < 0.001 vs. MCT + veh, ^##^ *p* < 0.01 vs. MCT + veh, ^#^ *p* < 0.05 vs. MCT + veh, ANOVA with Tukey’s post hoc test. Veh, vehicle; Mac, macitentan; Pip, Pipersentan; MWT, medial wall thickness; RVHI, right ventricular hypertrophy index.

Pipersentan at increasing concentrations was administered by oral gavage in MCT-induced PH rats, and macitentan was administered as a control (Figure 3A). The survival rates in each groups were 100%, 50%, 65%, 60%, 65%, 65%. There was significant weight loss in the MCT groups, but no difference was found among the pipersentan, macitentan and vehicle groups (Supplementary Figure S5A). In MCT-induced PH rats, both macitentan and pipersentan decreased RVSP and mPAP, the hemodynamic effects of pipersentan at a relatively lower dose (15 mg/kg) were similar to those of macitentan at 30 mg/kg, and the mPAP after pipersentan administration decreased in proportion to the dose (Figures 3B–D). Both pipersentan and macitentan at the dose of 30 mg/kg improved TAPSE. Meanwhile, pipersentan at the dose of 5 mg/kg could improved RVWT, which was comparable to macitentan at the dose of 30 mg/kg (Figures 3E–G). With regard to remodeling of pulmonary artery and right ventricle, the effects of pipersentan at a relatively lower dose (15 mg/kg) were similar to those of macitentan at 30 mg/kg, in attenuating the increases in MWT of pulmonary arterioles and RVHI of the heart (Figures 4A–C). These results suggested that pipersentan was comparable to macitentan at a relatively lower dose in terms of improvements in both pulmonary and cardiac remodeling and functions in MCT-induced PH rats.

### 3.7 Pipersentan Improved Cardiopulmonary Function and Remodeling in Hypoxia-Induced Pulmonary Hypertension Rats

**FIGURE 5:**
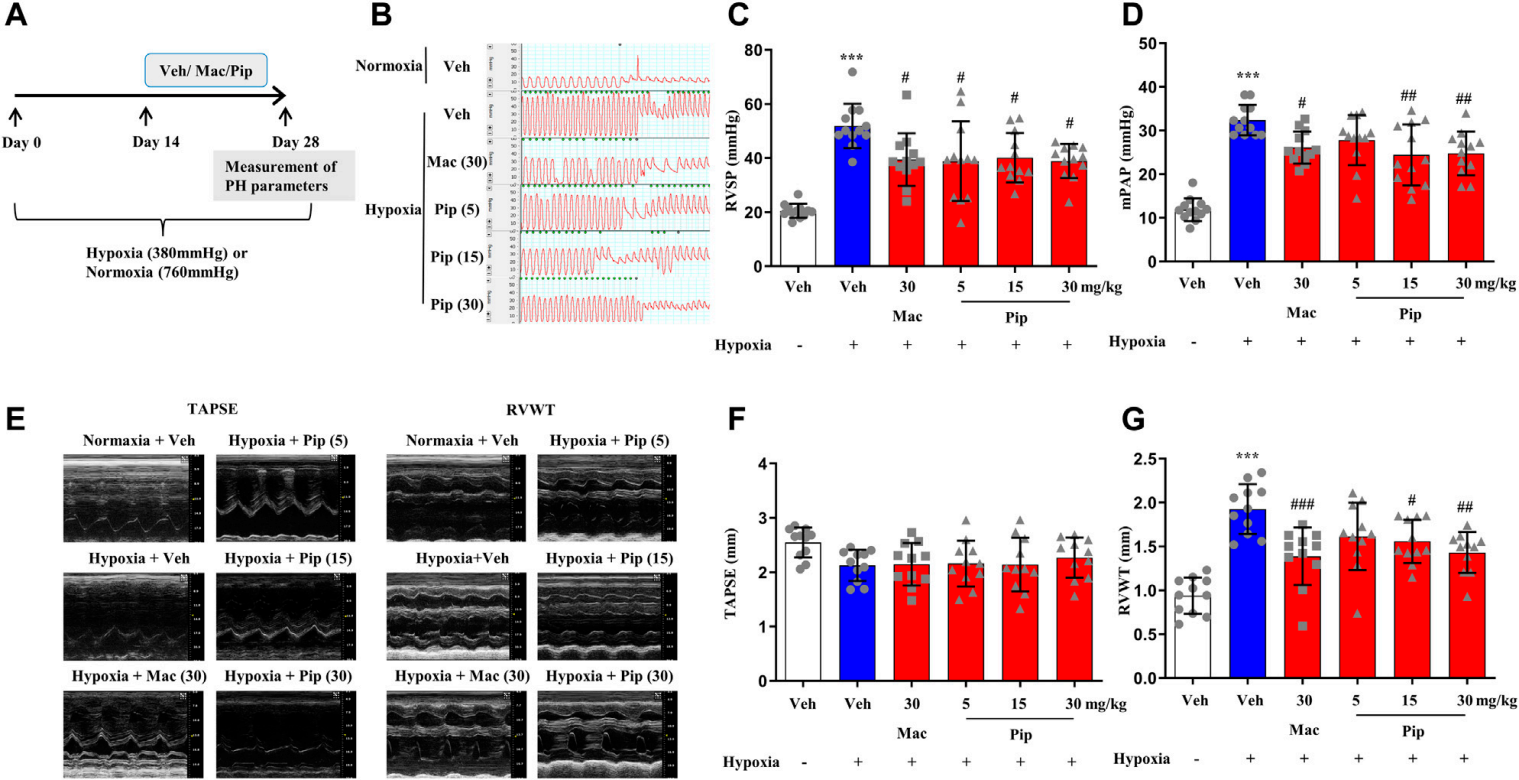
Pipersentan Improved Cardiopulmonary Function in Rats with Hypoxia-induced PH (*n* = 12) **(A)** Diagrammatic sketch of the animal protocol. Rats were housed in a hypobaric hypoxia chamber (with a barometric pressure of approximately 380 mmHg) or in normal air (with a barometric pressure of approximately 760 mmHg) for 28 days. Treatments by daily oral gavage started on day 14 and were continued for 2 weeks. Measurement of PH parameters was performed on day 28 **(B–D)** Hemodynamic measurements. **(B)** Representative images. **(C)** RVSP. **(D)** mPAP **(E–G)** UCG analyses. **(E)** Representative images. **(F)** TAPSE. **(G)** RVWT. The data are presented as the mean ± standard deviation, ^***^ *p* < 0.001 vs. veh, ^###^ *p* < 0.001 vs. Hypoxia + veh, ^##^ *p* < 0.01 vs. Hypoxia + veh, ^#^ *p* < 0.05 vs. Hypoxia + veh, ANOVA with Tukey’s post hoc test. Veh, vehicle; Mac, macitentan; Pip, Pipersentan; RVSP, right ventricular systolic pressure; mPAP, mean pulmonary artery pressure; UCG, ultrasonic cardiogram; TAPSE, tricuspid annular plane systolic excursion; RVWT, right ventricular wall thickness.

**FIGURE 6:**
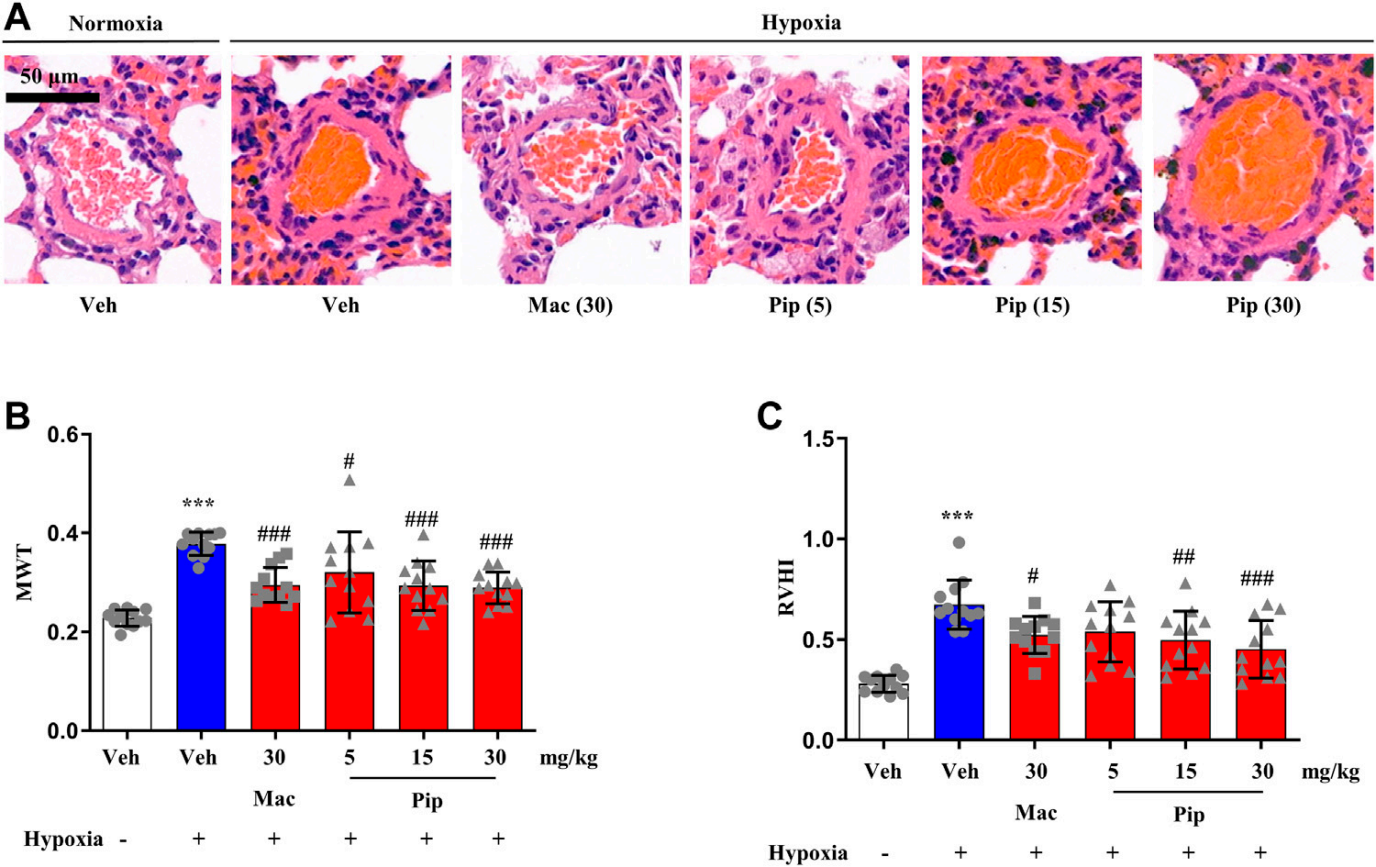
Pipersentan improved Cardiopulmonary Remodeling in Rats with Hypoxia-induced PH (*n* = 12). **(A,B)** Histopathological analysis. A Representative images of pulmonary arterioles (scale bars, 50m, Original magnification: 200). B MWT. **(C)** RVHI. The data are presented as the mean standard deviation, ^***^P < 0.001 vs veh, ^###^P < 0.001 vs MCT+veh, ^##^P < 0.01 vs MCT+veh, ^#^P < 0.05 vs MCT+veh, ANOVA with Tukeys post hoc test. Veh, vehicle; Mac, macitentan; Pip, Pipersentan; MWT, medial wall thickness; RVHI, right ventricular hypertrophy index.

Although ERAs are only approved in the treatment of PAH, the ET-1 signaling passway also plays one of the central roles in vasoconstriction and arterial remodeling in hypoxic lung. To explore whether pipersentan could improve cardiopulmonary function and remodeling in hypoxia-induced PH, pipersentan at increasing concentrations was administered by oral gavage, and macitentan was administered as a control (Figure 5A). Following oral gavage, the rats appeared healthy, and no treatment-related mortality was observed. There was significant weight loss in the hypoxia groups, but there was no difference among the pipersentan, macitentan and vehicle groups (Supplementary Figure S5B). Pipersentan significantly decreased RVSP, mPAP and RVWT in hypoxic rats at doses of 15 and 30 mg/kg, which was comparable to macitentan at the dose of 30 mg/kg (Figures 5B–G). At the relatively lower dose of 15 mg/kg, the effects in pulmonary arterial and cardiac remodeling were comparable to those of macitentan at the dose of 30 mg/kg (Figure 6). These results indicated that pipersentan was comparable to macitentan at a relatively lower dose in terms of improvements in both pulmonary remodeling and right ventricular hypertrophy in hypoxia-induced PH rats.

## 4 Discussion

ET-1 is a long-term vasoconstrictor in the human cardiovascular system. The binding of ET-1 with the endothelin receptors ET_A_ and ET_B_ on the surface of pulmonary vascular SMCs can induce vasoconstriction and promote cell division. Under pathological conditions, excessive production of ET-1 leads to continuous vasoconstriction; stimulates cell proliferation and migration, fibrosis, and vascular remodeling; and contributes to the development of PH (Eroglu et al., 2020). Macitentan is a dual-target antagonist of ET_A_ and ET_B_ receptors developed by Actelion, a Swiss company, and was approved by the US FDA in 2013 for treating PAH (Selej et al., 2015). Pipersentan has a similar chemical structure and the same mechanism of action as macitentan, which can competitively inhibit the binding of ET-1 to endothelin receptors, especially the ET_A_ receptor. Our study reveals the pharmacology of pipersentan, which showed good efficacy both *in vitro* and *in vivo*. This drug is intended to be used for ET-1 system activation-related PH.

The ERA bosentan was introduced as the first oral treatment for PAH. However, bosentan treatment is related to a dose-dependent increase of liver transaminase levels, which is attributed to inhibition of BSEP, while macitentan does not interfere with BSEP (Treiber et al., 2014). BSEP is an ATP-binding cassette transporter that mediates the elimination of bile salts in liver cells. Inhibition of BSEP delivered to the gastrointestinal tract through the bile duct will cause bile salt accumulation in liver cells, resulting in bile obstruction and liver injury. In our research, the inhibitory effects of pipersentan and macitentan on BSEP were assessed to provide a reference for the effect of pipersentan on liver bile salt delivery pumps. The IC_50_ values of pipersentan and macitentan for BSEP inhibition were 43.8 and 0.47 μM, respectively. The inhibitory activity of pipersentan on BSEP was 93 times weaker than that of macitentan, suggesting that pipersentan may reduce the risk of liver toxicity caused by ERAs in clinical trials.

Peripheral edema is more common and pronounced in patients treated with ET_A_ receptor-specific drugs than in those treated with dual ERAs (Zhang et al., 2019). Pipersentan showed high antagonistic activity against the ET_A_ receptor with an IC_50_ of 1.60 nM, almost equal to that of macitentan (IC_50_ of 0.52 nM). Pipersentan exhibited weak antagonistic activity against the ET_B_ receptor with an IC_50_ of 32.0 μM, which was much higher than that of macitentan (IC_50_ of 3.40 μM). Pipersentan is more selective for the ET_A_ receptor than macitentan and has greater potential to clinically reduce the incidence of peripheral edema toxicity as a dual ERA than ambrisentan with selectivity for the ET_A_ receptor.

In the panel of 24 GPCR cAMP modulation assays, the antagonistic effect of pipersentan on the ET_A_ receptor at 10 μM exhibited a 99.1% response rate, showing good repeatability together with the results of the binding and functional assays. The antagonistic effect of pipersentan on CNR1 showed a 72.6% response rate. The concentration of 10 μM was 6,250 times the IC_50_ for the ET_A_ receptor (1.6 nM), and CNR1 was mainly distributed in the brain. The experiment on the tissue distribution of the drug found that the rate of infiltration of the drug into the brain was very low (Supplementary Table S2). According to the tissue distribution results, the concentration of the drug in the rat brain was less than 100 nM at 10 mg/kg, which was far lower than the inhibitory concentration for CNR1. Therefore, no obvious off-target effect was observed under the measured conditions.

One of the most commonly employed animal models of PAH is established with a single subcutaneous administration of MCT. In this model, pipersentan at lower doses of 15 mg/kg had similar pharmacodynamic effects as macitentan at 30 mg/kg in improving both pulmonary arterial pressure and remodeling, as well as cardiac function and hypertrophy. Long-term exposure to global hypoxia can also lead to PH. The role of ET-1 in vasoconstriction and arterial remodeling in hypoxic lung has received special attention in recent years (Kylhammar and Rådegran, 2017). Preclinical studies have revealed the beneficial effects of ERA treatment in ameliorating hypoxia-induced PH (Yuyama et al., 2005). In our study, pipersentan (15 mg/kg) and macitentan (30 mg/kg) both lowered cardiopulmonary pressure and attenuated pulmonary arterial remodeling and right ventricular hypertrophy, suggesting extensive application potential for ERAs.

Epidemiological data on PH, including PAH, are lacking. Current estimates suggest that PH affects about 1% of the global population, and this rate can increase by up to 10% in people over 65 years old (Hoeper et al., 2016). Our discovery of pipersentan will provide assistance in the treatment of PH. However, there are also limitations. On one hand, there have been no “head-to-head” clinical trials of the three existing ERAs, bosentan, ambrisentan, and macitentan (Rivera-Lebron and Risbano, 2017), and no clinical data have demonstrated which ERA is superior to another. Although our data showed that pipersentan might reduce the risk of liver toxicity than macitentan, decrease the incidence of peripheral edema than ambrisentan, and improve cardiopulmonary remodeling and function at a relatively lower dose than macitentan, these results should be investigated for further studies, especially when it is to be prescribed to patients. The pharmacodynamic differences, drug interactions and side effects of these drugs should be considered carefully. On the other hand, ERAs are only recommend in the treatment of PAH (group-1 of PH) at present. Although ERAs have been shown to improve exercise capacity during hypoxia, there are no reliable data from randomized controlled trials to show that they should be used in patients with PH caused by chronic lung disease (group-3). In our study, pipersentan could significantly decreased RVSP and mPAP and attenuated the increases in RVWT, RVHI and MWT in hypoxic rats, but it still needs much more protocols to investigate the effect of pipersentan on other groups of PH, specifically group-3.

## Data Availability

The original contributions presented in the study are included in the article/Supplementary Material, further inquiries can be directed to the corresponding author.

## Abbreviations

PAH = pulmonary arterial hypertension, ERAs = endothelin receptor antagonists, ET-1 = endothelin-1, PH = pulmonary hypertension, SMCs = smooth muscle cells, ECs = endothelial cells, CHO = Chinese hamster ovary, BSA = bovine serum albumin, K-N-MC = human neuroepithelioma, hPASMCs = human pulmonary artery smooth muscle cells, RFU = relative fluorescence unit, RLU = relative light unit, BSEP = bile salt export pump, DMSO = dimethyl sulfoxide, LDH = lactate dehydrogenase, MCT = monocrotaline, TAPSE = tricuspid annular plane systolic excursion, RVWT = right ventricular wall thickness, mPAP = mean pulmonary artery pressure, RV = right ventricle, LV = left ventricle, MWT = medial wall thickness, HE = hematoxylin-eosin, CNR1 = cannabinoid receptor 1.
