# Supplementary material for: Pipersentan: A De Novo Synthetic Endothelin Receptor Antagonist that Inhibits Monocrotaline- and Hypoxia-Induced Pulmonary Hypertension
Source: Front Pharmacol. 2022 Jun 20;13:920222. doi: 10.3389/fphar.2022.920222 (PMC9251115; doi:10.3389/fphar.2022.920222)

**Supplementary Figures and Tables**


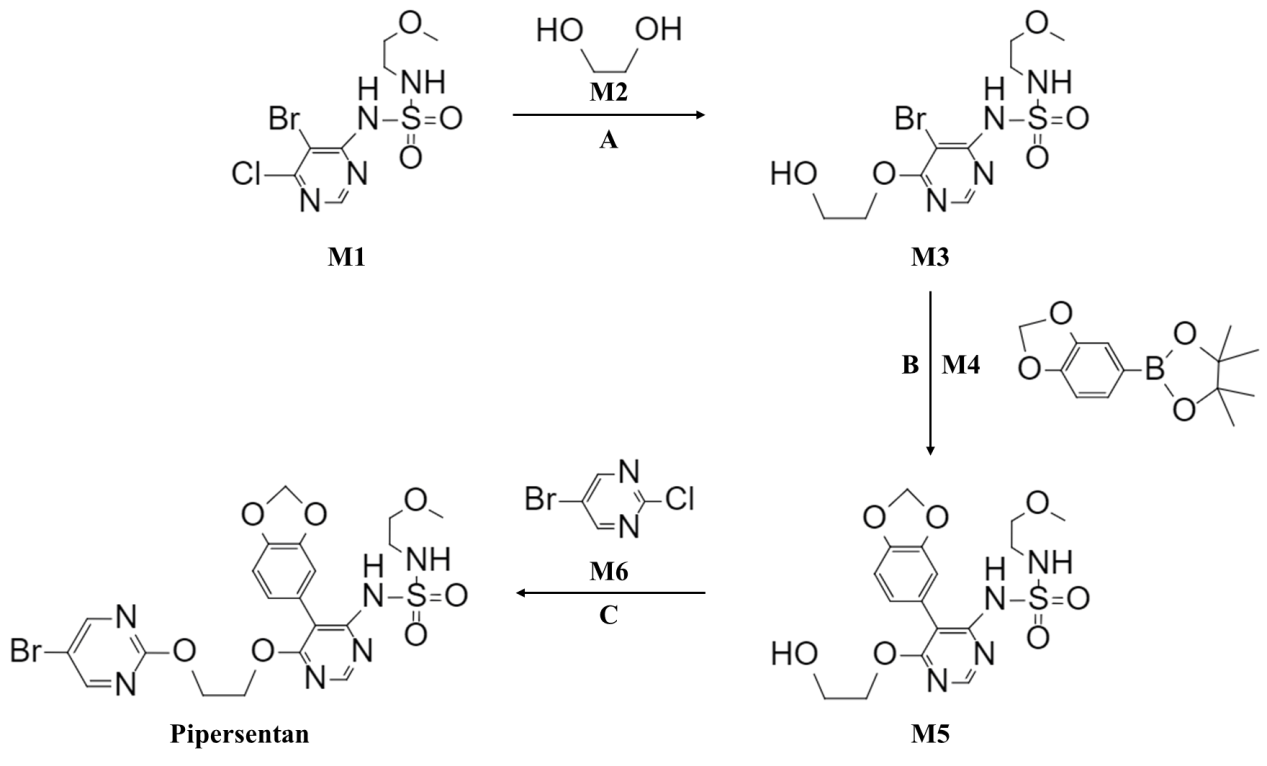


**Supplementary Figure S1. Chemical Synthesis of Pipersentan.**

1. t-BuOK. **(B)** Pd(dppf)Cl_2_, KOH, EtOH, H_2_O. **(C)** CS_2_CO_3_, THF. Please consult the authors for details.


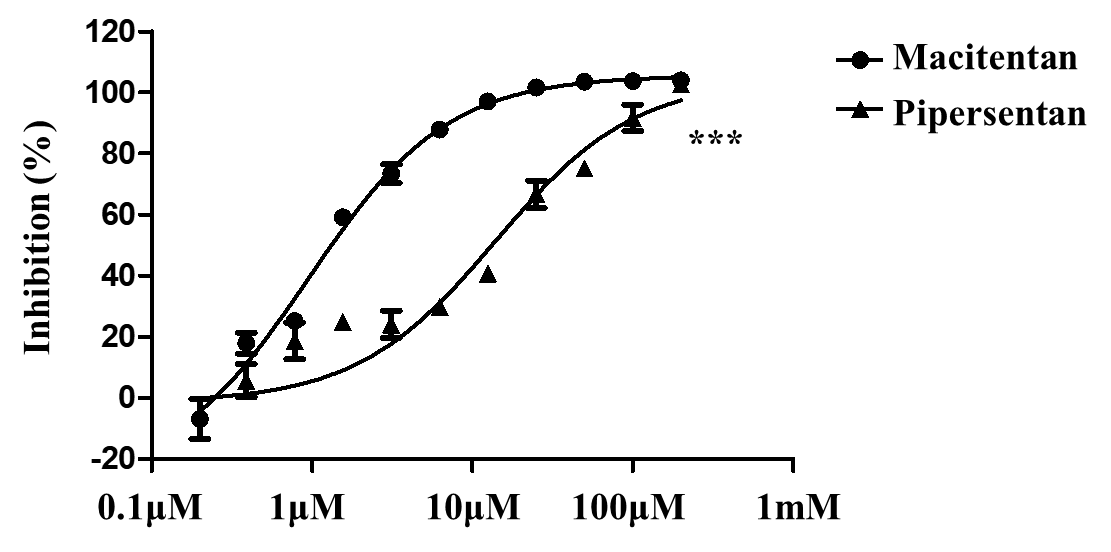


**Supplementary Figure S2**. Inhibition Assay of BSEP (n = 3).

The IC_50_ of Macitentan and Pipersentan on BESP inhibition was 0.47±0.02 nM and 43.8±7.95 nM. ^***^ P<0.001 vs Macitentan, unpaired Student's t-tests.


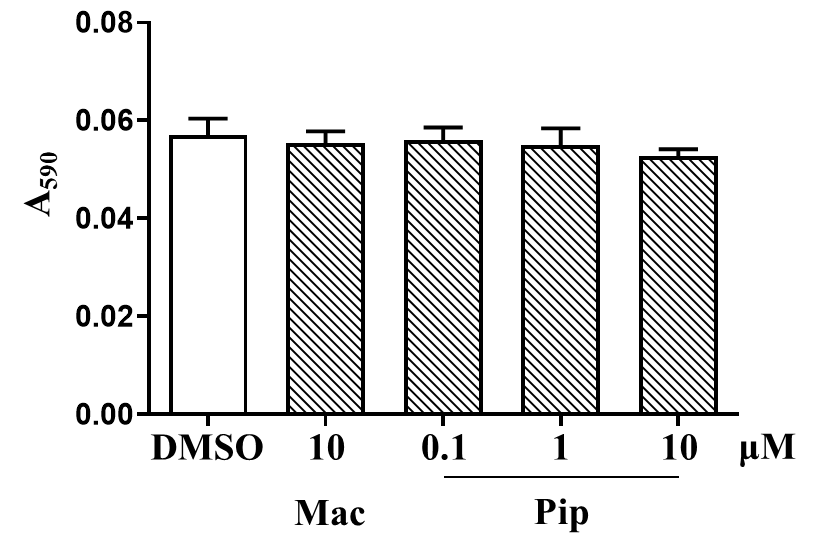


**Supplementary Figure S3.** Transwell migration assay for Macitentan and Pipersentan without ET-1 exposure (n = 3).


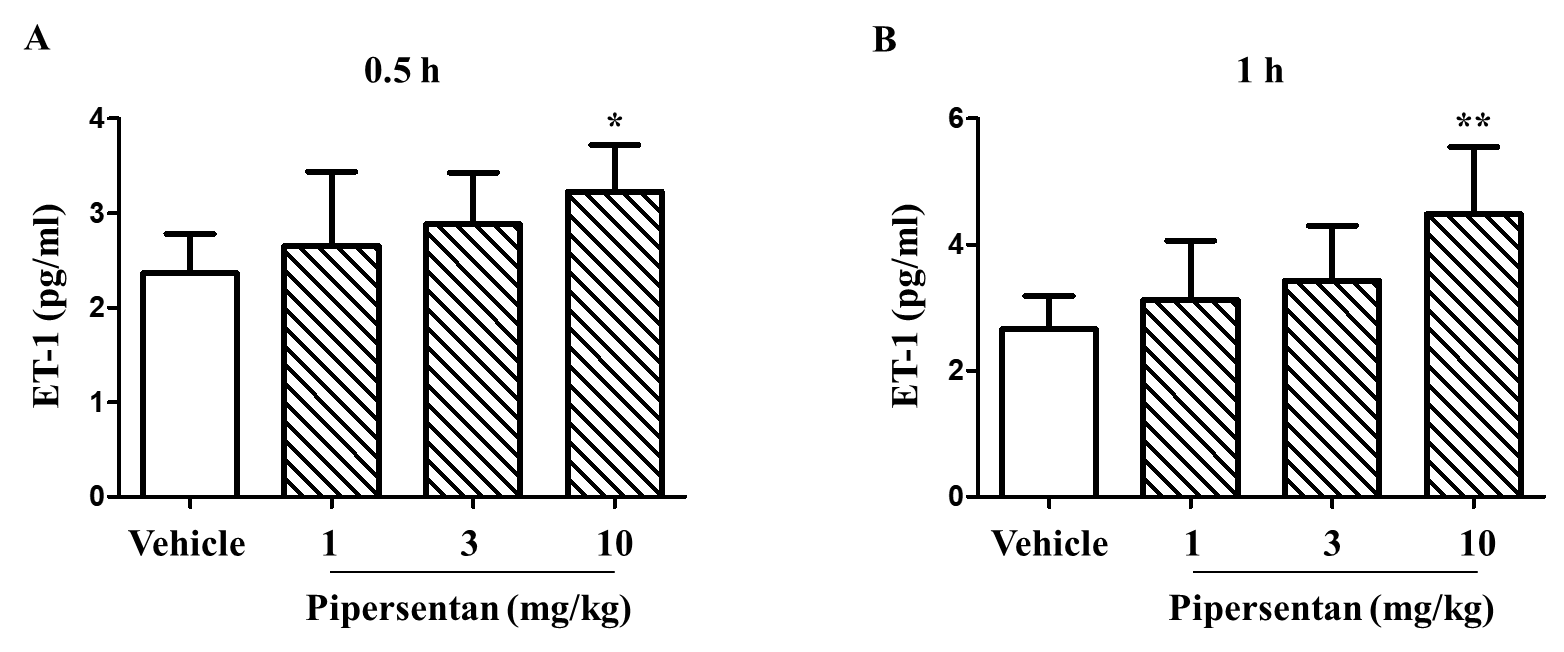


**Supplementary Figure S4.** ET-1 Plasma Concentrations in Rats at 0.5 h and 1 h after Oral Administration of Pipersentan with Increasing Concentrations (1, 3, 10 mg/kg) or Vehicle (n = 6). ^#^ P<0.05 vs Vehicle, ANOVA with Tukey’s post hoc test.


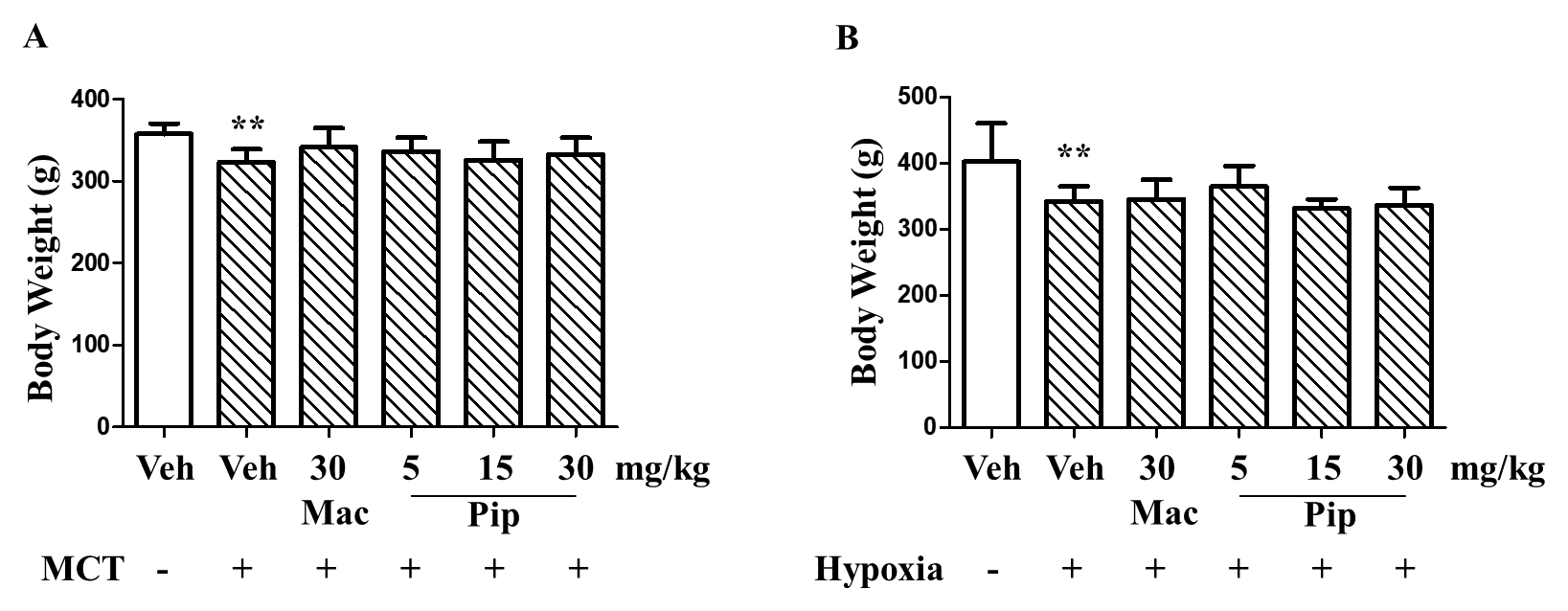


**Supplementary Figure S5.** Body Weights of Rats with MCT-Induced PH and Hypoxia-Induced PH (n = 12, 10, 13, 12, 13 and 13 for each group).^**^ P<0.01 vs Vehicle/-, ANOVA with Tukey’s post hoc test.

**Supplementary Table S1**. Binding Assays for cAMP-Related GPCRs.


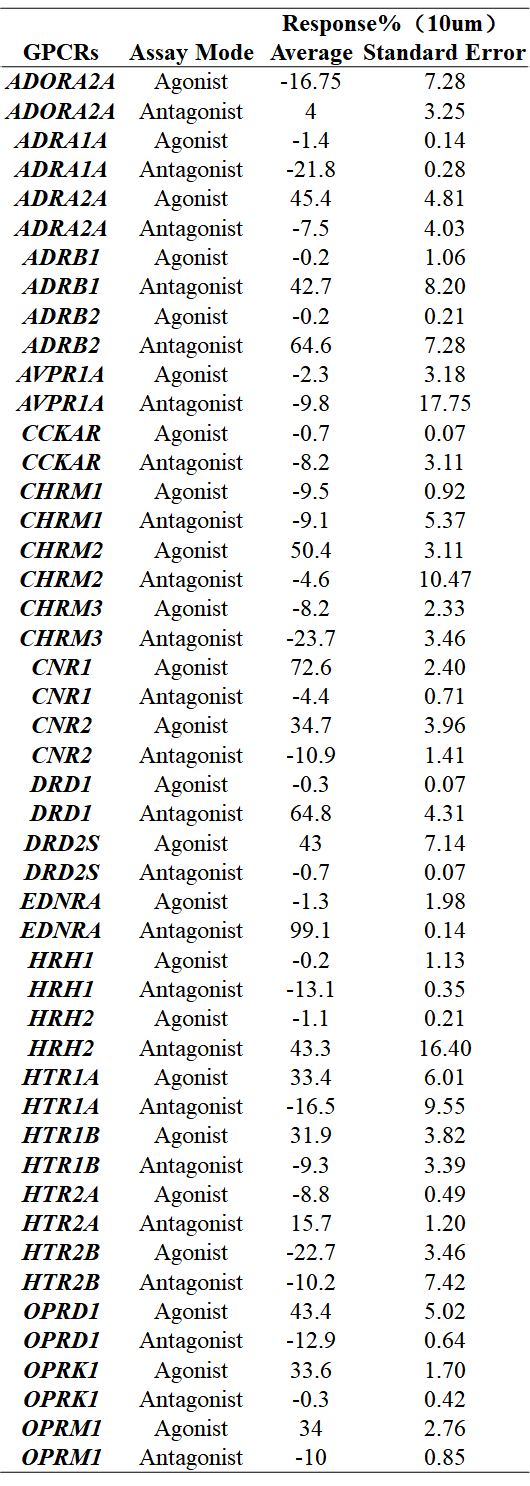
**Supplementary Table S2.** Total radioactivity in whole blood, plasma and tissues of SD rats at different time points after a single intragastric administration of ^[14C]^pipersentan at 3 mg/100 Ci/kg (n = 6, 3 males and 3 females).


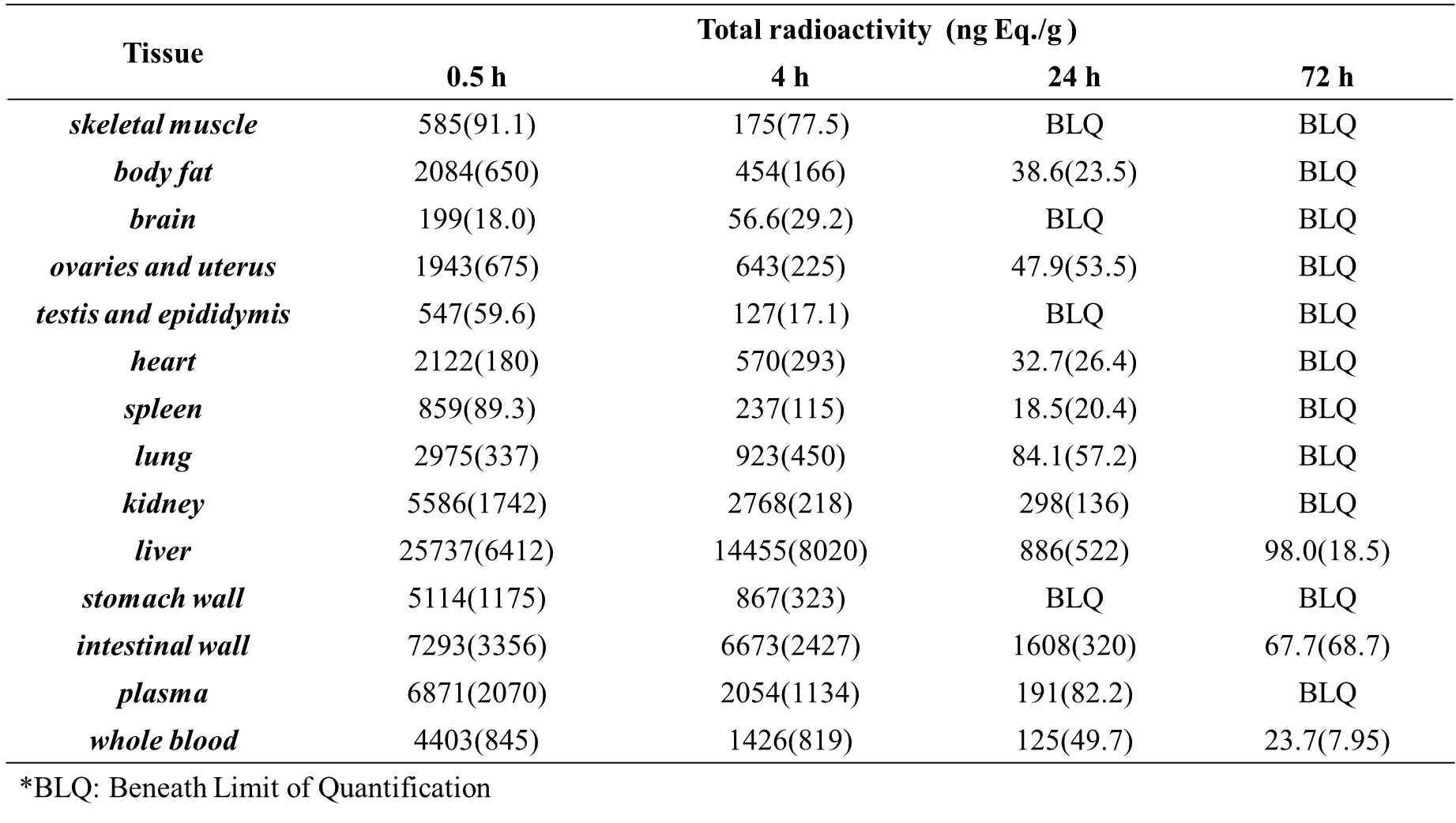

Supplement: Supplementary file 1 [file Table1.docx]
